# Modifiable factors to achieve target blood pressure in hypertensive participants

**DOI:** 10.1038/s41440-025-02134-x

**Published:** 2025-02-19

**Authors:** Sachiko Tanaka-Mizuno, Fumiko Nakatsu, Shunsuke Eguchi, Kazuma Iekushi, Hironori Nakagami

**Affiliations:** 1 Laboratory of Epidemiology and Prevention, Kobe Pharmaceutical University, Kobe, Japan; 2 Medical Affairs, Novartis Pharma K.K., Tokyo, Japan; 3 Department of Health Development and Medicine, Osaka University Graduate School of Medicine, Osaka, Japan

**Keywords:** Achievement, Hypertension, Prevention, Specific health checkups, Target blood pressure

## Abstract

The management of hypertension is one of the most important public health issues. Many patients with untreated hypertension in Japan require urgent treatment. This retrospective cohort study in Hiratsuka city aimed to evaluate the proportion of participants achieving target blood pressure and identify modifiable factors affecting the achievement. We retrospectively analyzed data from a merged database of claims, specific health checkup (SHC), and national health insurance data in Hiratsuka City, Japan, from June 2016 to March 2023. The study participants were adults aged 40–74 years without a history of hypertension treatment and with blood pressure ≥140/90 mmHg at SHC. The primary outcome was the achievement of target blood pressure <140/90 mmHg at the next SHC. Furthermore, multivariable logistic regression was performed to explore factors influencing the achievement. Of 5428 participants, 43.6% were female. The median age was 69 years, and 58.4% (95% confidence interval 57.1–59.7) achieved target blood pressure <140/90 mmHg. Multivariable logistic regression results showed that achievement of target blood pressure was associated with younger age (50–69 years), mild hypertension (grade I), no hypertension at the previous SHC, no record of SHC in the previous year, and willingness to improve lifestyle. One-third of people reported that their hypertension at SHCs failed to achieve target blood pressure. For community-level hypertension management, people who have the influencing factors must be educated by public health nurses, which might be effective for lifestyle improvement. Additionally, the elderly and people with persistent hypertension or severe hypertension should seek medical advice.

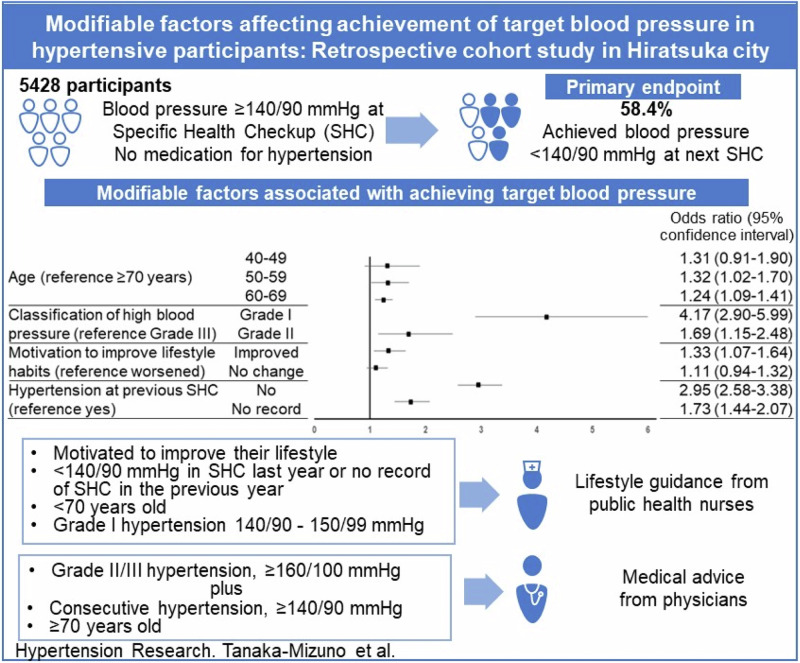

## Introduction

Hypertension is a significant risk factor for cardiovascular (CV) events and all-cause mortality [1, 2]. Globally, an estimated 1.28 billion adults aged 30–79 years have hypertension, with most (two-thirds) of them living in low- and middle-income countries [3]. The prevalence of hypertension is increasing worldwide, and an estimated 29% of the world’s population will have hypertension by 2025 [4]. Hypertension awareness and treatment rates are increasing in high-income countries, whereas they are lower in Japan [5]. In fact, 43 million people have hypertension in Japan, with 44% of them having untreated hypertension [6].

Some people are unaware of their hypertension status (16% of people in the United States [US] [7]; 16%, Canada [8]; and 33%, Japan [6]), whereas some people are aware of their hypertension status but remain untreated (7% of people in the US; 4%, Canada; and 11%, Japan) [6–8]. To address this issue, it is essential to identify people who have undiagnosed or untreated hypertension and initiate lifestyle modification and therapeutic intervention to normalize their blood pressure (BP) as early as possible.

In Japan, medical insurers are mandated to conduct annual specific health checkups (SHCs) and specific health guidance (SHG) for insured people and their dependents aged ≥40 and <75 years, according to the Securing Medical Care for the Elderly Act, which was revised in 2008 as part of the healthcare system reform. SHCs help prevent risk factors for CV events by detecting metabolic syndrome at an early stage and providing SHG to patients to improve exercise, dietary, and lifestyle habits. This study used SHC data from National Health Insurance (NHI) to examine the current status and characteristics of individuals with untreated or interrupted hypertension in Hiratsuka City. Previous SHC data recorded for Hiratsuka City revealed that ~52% of the recipients had a systolic BP (SBP) of ≥130 mmHg [9], which is higher than the national average of ~45.6% [10]. Furthermore, ~25% of the recipients had BP levels classified as stage I or higher, and ~43% remained untreated. Additionally, the standardized mortality ratios by disease in Hiratsuka City indicated a significantly higher incidence of intracerebral hemorrhage among males (16.1% increase over the national average) [9]. To improve population health outcomes, there is an urgent need for an effective approach to provide medical care and manage hypertension at the regional level.

This study aimed to assess the characteristics of participants with hypertension who were either untreated or had interrupted treatment, evaluate the proportion of participants who achieved the target BP (TBP), and explore the modifiable factors affecting the achievement.

Point of view

- Clinical relevance
  In the retrospective analysis of specific health checkup (SHC) and national health insurance data in Hiratsuka City, Japan, 58.4% of 5428 participants achieved target blood pressure <140/90 mmHg.
- Future direction
  For community-level hypertension management, education by public health nurses might be effective for lifestyle improvement, and the elderly and people with persistent hypertension or severe hypertension should also seek the medical advice.
- Consideration for the Asian population
  Since health management requires human resources, we should consider to provide a guidance based on priorities with clinical evidence. For the Asian population, the efficient guidance can be tailored to the characteristics of each individual in future.

## Methods

### Data source

This study used an NHI database in Hiratsuka City, Japan, to collect information on SHCs and medical claims from May 1, 2016, to March 31, 2023, and the list of people insured by the NHI in Hiratsuka City from October 1, 2017, to March 31, 2023. After integrating all databases and processing them into a format that ensured blinding of any specific individuals, the city permitted the research consortium of Osaka University and Novartis Pharma K. K. to use the database for analysis on the condition that the analysis results be returned in a form that could be used for city healthcare projects. The SHC database includes information on demographic characteristics, anthropometric measurements, laboratory test values, and the results of a self-administered questionnaire on lifestyle behaviors. The NHI database includes information on postal codes and the date on which the participants were insured and left out of the insurance. The claims database includes information on prescribed drugs and medical treatments.

### Study design

This was a retrospective cohort study of participants with untreated hypertension in Hiratsuka City that investigated the characteristics of the participants and the proportion who achieved TBP and explored the modifiable factors affecting the achievement.

Among Hiratsuka City NHI subscribers, eligible participants who had a record of ≥2 SHCs from June 1, 2016, to March 31, 2023, and met the eligibility criteria were identified. The index date is defined as the latest SHC date on which the participant met the eligibility criteria. Participants who met the following criteria were included in the study: (1) those aged 40–74 years, which is the target age for SHC; (2) those with ≥2 SHC results within 3 years of the study period and whose BP ≥ 140/90 mmHg, indicating hypertension [3, 6] in at least the second most recent result of the examination; (3) those whose record of the following SHC is available within 2 years and claims data between the index date and the following SHC are available; (4) those who have been enrolled in the NHI system at least 6 months before the index date. Participants with a record of hypertension-related injury, disease, or prescription of antihypertensive medication before the index date were excluded. The index date spanned from April 1, 2018, to March 31, 2022, because confirmation of the claims database (from October 1, 2017, to March 31, 2023) was required for 6 months before the index date, for the baseline period, for 1 year following the index date, and for the next SHCs. Therefore, the next SHC spanned from April 1, 2019, to March 31, 2023.

### Variables

We obtained information on demographic characteristics (age, sex, SBP, diastolic BP [DBP], and body mass index [BMI]) and the presence of metabolic disorders (diabetes mellitus and dyslipidemia). SBP and DBP at the index date were also categorized according to the Japanese Society of Hypertension Guidelines for the Management of Hypertension (JSH 2019) [6]: Grade I hypertension (SBP 140–159 mmHg and/or DBP 90–99 mmHg), Grade II hypertension (SBP 160–179 mmHg and/or DBP 100–109 mmHg), and Grade III hypertension (SBP ≥ 180 mmHg and/or DBP ≥ 110 mmHg).

We also extracted the records to assess for any history of CV events (heart disease and stroke), chronic kidney disease (CKD), and anemia; the status of hypertension at the previous SHC; participation in SHG; new onset of metabolic disorders and CKD, and occurrence of CV events.

The results of a self-administered questionnaire on lifestyle, personality, or motivation reflective characteristics (smoking status, exercise habits, physical activity, chewing status, eating speed, late-night eating, snacking, skipping breakfast, drinking status, alcohol consumption, sleep quality, interest in receiving health guidance, and motivation to improve lifestyle habits) were evaluated as risk factors. The items of these lifestyle characteristics at the index date were measured just before participants were identified as having hypertension. To assess modifiable behavior change, we evaluated changes in responses between the index date and the next SHCs and stratified them into “improved,” “no change,” and “worsened.”

We categorized Hiratsuka City into five residential areas to evaluate the impact of the living environment: area A: located near the central train station, in a central residential area with relatively many commercial and public facilities, but not as many as B. There are also many medical institutions; area B: located near the train station, with a high number of commercial and public facilities as well as medical institutions. Convenient transportation is also available; area C: located in the suburbs, with a high number of commercial facilities and fewer medical institutions. However, the medical institutions are mainly located in the commercial area; area D: located far away from the city center, there are relatively many commercial and public facilities, but not as many as B. Transportation is inconvenient, and there are few medical institutions; area E: located far away from the city center, with few commercial facilities. Transportation is inconvenient, and there are few medical institutions.

More detailed information on all variables is presented in Table S1. Diseases and drugs were defined according to the International Classification of Diseases, 10th revision (ICD-10), version for 2013, and standardized drug codes managed by Health Insurance Claims Review & Reimbursement Service, which lists individual prescription drugs in Japan, based on the versions from December 8, 2022, were used, and they were further checked for appropriateness by the authors.

### Outcomes

**Fig. 1:**
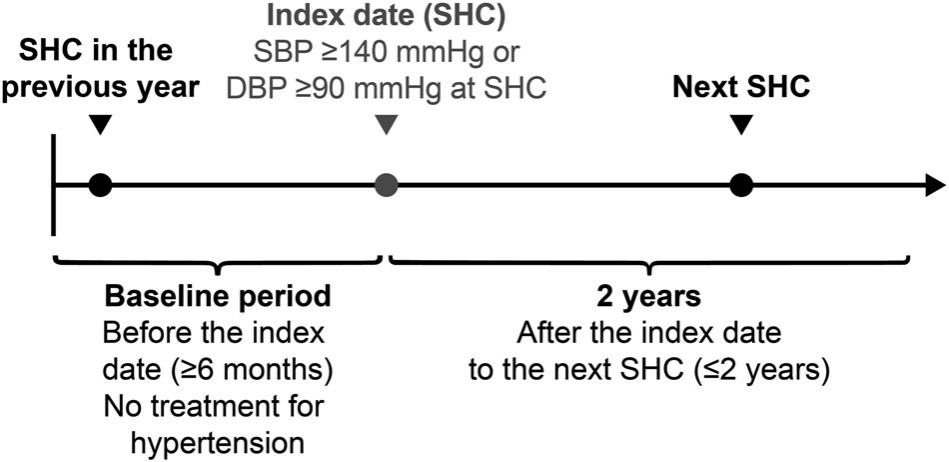
Study design. Abbreviations: DBP diastolic blood pressure, SBP systolic blood pressure, SHC specific health checkup

The primary outcome was the achievement of SBP < 140 mmHg and DBP < 90 mmHg (achievement of TBP < 140/90 mmHg) at the next SHC in participants with hypertension who had their BP detected at the SHC, which is the index date (Fig. 1). The secondary outcome was achieving another TBP, with SBP < 130 mmHg and DBP < 80 mmHg (achievement of TBP < 130/80 mmHg). The other outcome measure was evaluating the characteristics of participants who did or did not achieve TBP.

### Statistical analysis

Data of participant characteristics, behaviors, lifestyle, history of diseases, and medical history before or at the index date were described as summary statistics of numbers and percentages. Changes in the measurement of the self-administered questionnaire between the index date and the next SHC were also described. Achievement of TBP < 140/90 mmHg or TBP < 130/80 mmHg at the next SHC was descriptively summarized as numbers and proportions.

Logistic regression analysis was performed to estimate odds ratios (ORs) and 95% confidence intervals (CIs), with the achievement proportion of the TBP as the outcome. Univariable logistic regression was performed, followed by a multivariable logistic regression, including variables with *p* values for parameter estimates of less than 0.05 in the univariable analysis. Data from individuals with missing values will not be used, and only participants with a complete set of variables needed for this study were included (complete case analysis). Statistical analysis was performed with Python 3.8.10.

### Ethics statement

This study was conducted according to the Ethical Guidelines for Medical and Health Research Involving Human Subjects (Ministry of Health, Labor and Welfare. Ethical Guidelines for Medical and Health Research Involving Human Subjects; available from https://www.mhlw.go.jp/file/06-Seisakujouhou-10600000-Daijinkanboukouseikagakuka/0000080278.pdf. 2023), and the Ethical Review Board Osaka University Hospital approved the research protocol (No. 23009). The committee approved the opt-out consent method. The opt-out document was posted at the Hiratsuka City Office and Osaka University.

## Results

**Fig. 2:**
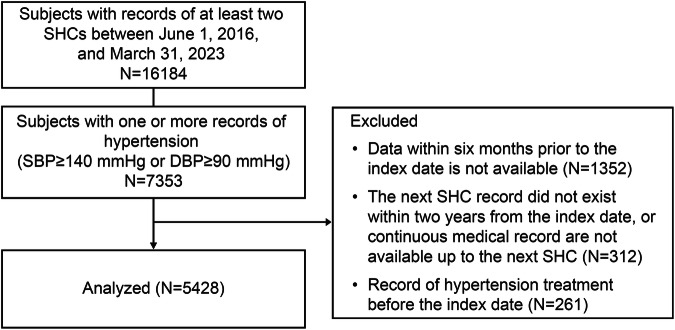
Study flowchart. Abbreviations: DBP diastolic blood pressure, SBP systolic blood pressure, SHC specific health checkup

Overall, 5428 of 16,184 participants with at least 2 SHC records between June 1, 2016, and March 31, 2023, were finally included in the analysis after applying the eligibility criteria (Fig. 2).

Table 1 shows the summary statistics of the participants’ baseline and clinical characteristics. Among the study participants, 43.6% were male. As for age, 47.5% were aged 60–69 years and 42.5% were aged 70–74 years, accounting for most participants. The proportion of participants with grade I, grade II, and grade III hypertension was 80.5%, 16.1%, and 3.4%, respectively. At the index date, 44.7% of participants had exercise habits, 24.3% ate fast, and 27.4% had no intention to improve their lifestyle. Of those who had no exercise habits and those who ate quickly, only up to 10% had improved after the index date to the next SHC (10.1% of participants with exercise habits, 7.1% of those who ate quickly). However, more participants showed improvement in lifestyle modification (17.4%; Table S2). The most common history of past disease was dyslipidemia, observed in 34.8% of participants, followed by chronic kidney disease/renal failure in 20.3%, anemia in 12.5%, and heart disease in 10.9%. Moreover, 43.6% of participants had high BP, as recorded at their previous SHC, 41.2% did not have high BP, and 15.2% had no record of a previous SHC in the past year.

**Table 1:** Summary of baseline and clinical characteristics (*N* = 5428)

Extraction period	Characteristics		*n* (%)
Index date	Sex	Male	2368 (43.6)
	Age, years	40–49	170 (3.1)
		50–59	376 (6.9)
		60–69	2574 (47.5)
		70–74	2303 (42.5)
	BMI, kg/m^2^	<25	3783 (69.7)
		25 to <30	1361 (25.1)
		≥30	284 (5.2)
	Classification of high BP^a^	Grade I	4368 (80.5)
		Grade II	874 (16.1)
		Grade III	186 (3.4)
	Residential area^b^	A	851 (15.8)
		B	1348 (25.0)
		C	834 (15.5)
		D	1086 (20.1)
		E	1274 (23.6)
	Smoking status	Yes^g^	624 (11.5)
	Exercise habits	Yes^h^	2422 (44.7)
	Physical activity	Yes^i^	2744 (50.7)
	Walking speed	Fast^j^	2827 (52.4)
	Chewing condition	Able to chew anything	4411 (81.5)
		Have difficulties chewing in some areas	970 (17.9)
		Barely able to chew	30 (0.6)
	Eating speed^c^	Fast	1309 (24.3)
	Late-night eating^d^	Yes	833 (15.4)
	Snacking^e^	Daily	971 (18.0)
		Occasionally	3231 (59.7)
		Hardly consume	1207 (22.3)
	Skipping breakfast^f^	Yes	444 (8.2)
	Drinking status	Daily	1398 (25.8)
		Occasionally	1281 (23.7)
		Hardly consume (unable to drink)	2731 (50.5)
	Alcohol consumption	<180 ml	4036 (74.6)
		180 to <360 ml	908 (16.8)
		360 to <540 ml	372 (6.9)
		≥540 ml	92 (1.7)
	Sleep quality	Enough	4967 (91.8)
	Interest in receiving health guidance	Yes	1939 (35.9)
	Motivation to improve lifestyle habits	None	1474 (27.4)
		Will start soon	2304 (42.8)
		Have been engaged already	1610 (29.9)
Baseline	History of diseases	Diabetes	524 (9.7)
		Dyslipidemia	1891 (34.8)
		Stroke	366 (6.7)
		Heart disease	589 (10.9)
		CKD/renal failure	1102 (20.3)
		Anemia	676 (12.5)
Previous SHC	Hypertension at the previous SHC	Yes	2366 (43.6)
		No	2236 (41.2)
		No record	826 (15.2)

Data are presented as numbers and percentages

*BMI* body mass index, *BP* blood pressure, *CKD* chronic kidney disease, *SHC* specific health checkup

^a^Classification of high BP: grade I, SBP 140–159 mmHg and/or DBP 90–99 mmHg; grade II, SBP 160–179 mmHg and/or DBP 100–109 mmHg; grade III, SBP ≥ 180 mmHg and/or DBP ≥ 110 mmHg

^b^Residential area A, located near the central train station, in a central residential area with relatively many commercial and public facilities, but not as many as B. There are also many medical institutions; B, located near the train station, with a high number of commercial and public facilities, as well as medical institutions. Convenient transportation is also available; C, located in the suburbs, with a high number of commercial facilities and fewer medical institutions. However, the medical institutions are mainly located in the commercial area; D, located far away from the city center, with relatively many commercial and public facilities, but not as many as B. Transportation is inconvenient, and there are few medical institutions; E, located far away from the city center, with few commercial facilities. Transportation is inconvenient, and there are few medical institutions

^c^Participants were asked the following questions in the questionnaire, “Do you eat faster than others?”

^d^Participants were asked the following questions in the questionnaire, “Do you have dinner within 2 h before going to bed more than 3 times a week?”

^e^Participants were asked the following questions in the questionnaire, “Do you consume snacks or sweet drinks in addition to the three meals of breakfast, lunch, and dinner?”

^f^Participants were asked the following questions in the questionnaire, “Do you skip breakfast at least 3 times a week?”

^g^Participants were asked the following questions in the questionnaire, “Have you smoked at least 100 cigarettes during your entire life or for at least 6 months, and do you smoke in the past month?”

^h^Participants were asked the following questions in the questionnaire, “Do you exercise lightly sweating for at least 30 min at a time, at least 2 days a week, for at least 1 year?”

^i^Participants were asked the following questions in the questionnaire, “Do you walk or perform in equivalent physical activity in your daily life for at least 1 h per day?”

^j^Participants were asked the following questions in the questionnaire, “Do you walk faster than people of about the same age and gender?”

Of the 5428 participants, 58.4% (95% CI 57.1–59.7%) achieved TBP < 140/90 mmHg (Table 2). However, 18.1% (95% CI 17.0–19.1%) achieved TBP, with BP < 130/80 mmHg, and this proportion was as low as one-third of that for the achievement proportion of TBP < 140/90 mmHg. Interestingly, 2468 (42.6%) participants were taken antihypertensive medicines continuously after the SHC and 2960 (54.4%) participants were not. The achievement rate of TBP < 140/90 mmHg of those who took the medications was 61.4% and of those who did not take the medications was 55.9%.

**Table 2:** Blood pressure changes in participants with hypertension

Target blood pressure	Number of achievements (n)	Achievement proportion (%)	95% CI
SBP < 140 and DBP < 90 mmHg	3171	58.4	57.1–59.7
SBP < 130 and DBP < 80 mmHg	980	18.1	17.0–19.1

Data are presented as numbers and percentages

*CI* confidence interval, *DBP* diastolic blood pressure, *SBP* systolic blood pressure

Based on the results of the univariable logistic regression to explore each risk factor related to the achievement of TBP < 140/90 mmHg (Fig. S1), age, BMI, classification of high BP, residential area, smoking status, exercise habits, physical activity, eating habits, sleeping quality, interest in receiving SHG, motivation to improve lifestyle habits, pre-existing diseases, hypertension at the previous SHC, new onset of metabolic syndromes and CKD, and CV events were included in the multivariable logistic regression.

**Fig. 3:**
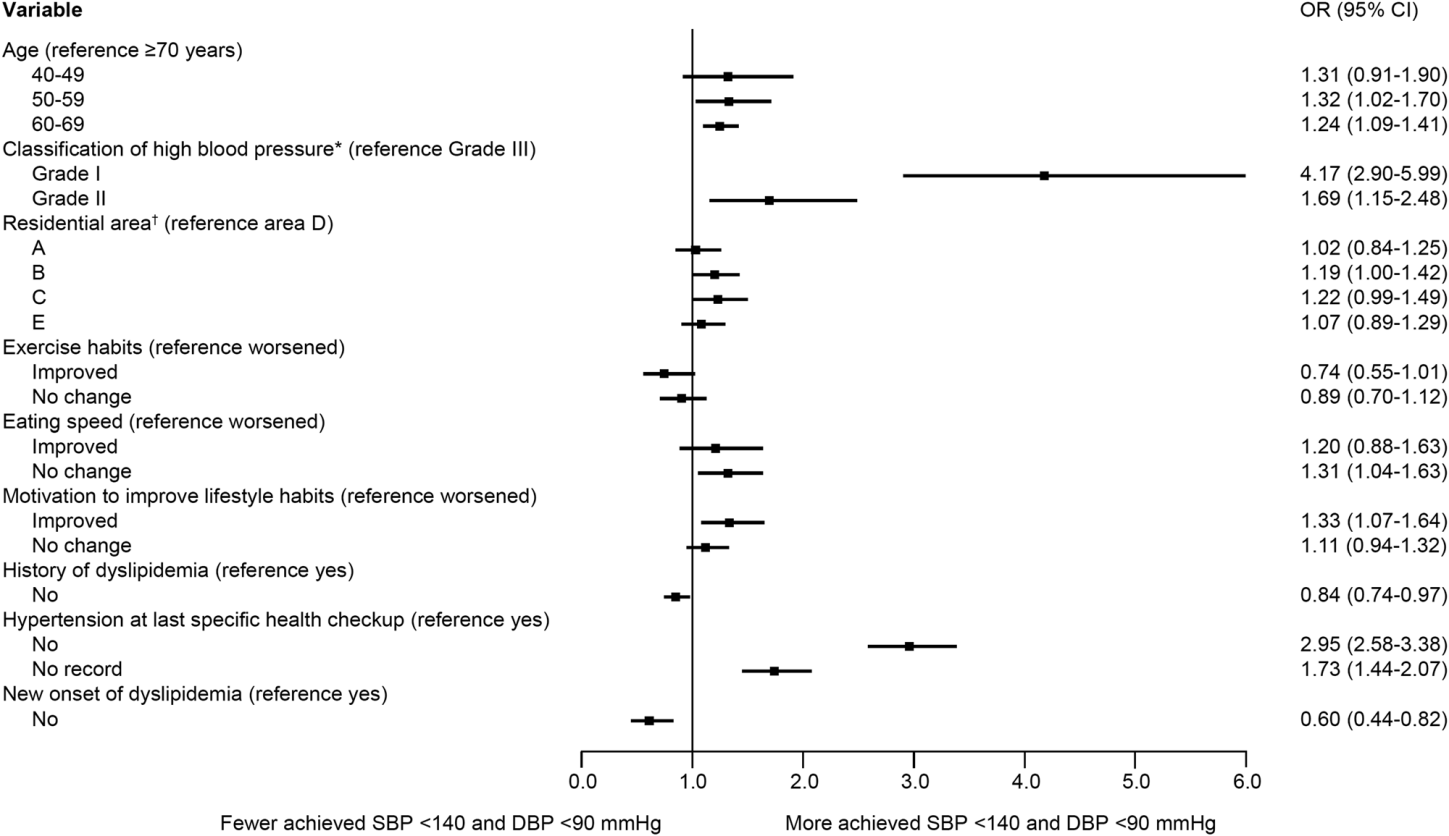
Exceptional results of multivariable logistic regression analysis of factors affecting achievement of target blood pressure <140/90 mmHg. The adjusted odds ratios for achieving TBP < 140/90 mmHg were estimated using the multivariable logistic regression model including the following variables: sex; age; BMI; classification of high BP; residential area; history of diabetes, dyslipidemia, stoke, heart disease, CKD/renal failure, or anemia; hypertension at the previous SHC; new onset of diabetes, dyslipidemia, or CKD; and change in score after the index date to the next SHC for the following variables: smoking status, weight gain since the age of 20 years, exercise habits, physical activity, walking speed, chewing condition, eating speed, snacking, skipping breakfast, drinking status, alcohol consumption, sleep quality, interest in receiving SHG, motivation to improve lifestyle habits, and lifestyle score. *Classification of high BP: grade I, SBP 140–159 mmHg and/or DBP 90–99 mmHg; grade II, SBP 160–179 mmHg and/or DBP 100–109 mmHg; grade III, SBP 180 mmHg and above and/or DBP 110 mmHg and above. ^†^Residential area A, located near the central train station, in a central residential area with relatively many commercial and public facilities, but not as many as B. There are also many medical institutions; B, located near the train station, with a high number of commercial and public facilities, as well as medical institutions. Convenient transportation is also available; C, located in the suburbs, with a high number of commercial facilities and fewer medical institutions. However, the medical institutions are mainly located in the commercial area; D, located far away from the city center, with relatively many commercial and public facilities, but not as many as B. Transportation is inconvenient, and there are few medical institutions; E, located far away from the city center, with few commercial facilities. Transportation is inconvenient, and there are few medical institutions. Abbreviations: BP blood pressure, BMI body mass index, CKD chronic kidney disease, DBP diastolic blood pressure, OR odds ratio, SBP systolic blood pressure, SHC specific health checkup, SHG specific health guidance, TBP target blood pressure

Figure 3 (the results of all variables are presented in Fig. S2) shows the results of multivariable logistic regression. Factors with an OR of ≥1 were in the direction of the increasing achievement of TBP < 140/90 mmHg, and factors with an OR of ≤1 were in the direction of lowering achievement of TBP < 140/90 mmHg. Compared with the 70–74 years age group, the ORs (95% CIs) for the 50–59 years age group and the 60–69 years age group were 1.32 (1.02–1.70) and 1.24 (1.09–1.41), respectively, and the 50–69 age category shows an increase in target achievement proportion. Compared with grade III hypertension, grade I and grade II hypertension elevated the achievement of TBP < 140/90 mmHg (OR [95% CI]: 4.17 [2.90–5.99] and 1.69 [1.15–2.48], respectively). Compared with groups living in agricultural and industrial areas with fewer medical facilities at group D, groups living in areas near train stations with many medical facilities at group B and suburban residential areas with fewer medical facilities at group C contributed to improved BP control (OR [95% CI]: 1.19 [1.00–1.42] and 1.22 [0.99–1.49], respectively).

Regarding the improvement and no change in the lifestyle factors compared with the worsening of the factors, exercise habits, eating speed, sleep quality, and motivation to improve lifestyle habits were associated with the achievement of TBP < 140/90 mmHg (OR [95% CI]: 0.74 [0.55–1.01] and 0.89 [0.70–1.12] vs. 1.20 [0.88–1.63], 1.31 [1.04–1.63], 1.33 [1.07–1.64], and 1.11 [0.94–1.32], respectively). Regarding the history of hypertension at the previous SHC, compared with those with hypertension at the previous SHC, those without hypertension or no record of the previous SHC were associated with the achievement of TBP < 140/90 mmHg (OR [95% CI]: 2.95 [2.58–3.38] and 1.73 [1.44–2.07]). History of dyslipidemia and new onset of dyslipidemia decreased the achievement proportion with OR (95% CI): 0.84 (0.74–0.97) and 0.60 (0.44–0.82), respectively.

## Discussion

In the primary outcome analysis of this study, the proportion of participants with hypertension (*n* = 5428) who achieved TBP < 140/90 mmHg at the next SHC was 58%, which is a higher achievement proportion than that reported previously. In a previous analysis of similar participants with hypertension, 817 of the 2785 men (29%) and 217 of 790 women (27%) who were diagnosed with hypertension at SHCs had improvement to TBP < 140/90 mmHg in the following year [11]. Another study reported that the proportion of participants with improved BP was 52% for those on antihypertensive medication and 37% for those not on antihypertensive medication [12], and the achievement proportion in this study was higher than that reported previously. While these differences may be due to the region or year period among studies, this study had a larger number of participants and an age structure more like that of the general population than that reported in previous studies, which may reflect a more real-world setting.

In the secondary outcome analysis, the achievement proportion of participants with TBP < 130/80 mmHg was only 18%, which is significantly lower than that of TBP < 140/90 mmHg. Although high BP is defined as SBP of 130-140 mmHg and DBP of 80-90 mmHg, this result strongly suggests the need for stronger awareness of normal BP.

Further analysis revealed factors that contribute to achieving TBP < 140/90 mmHg. The results of multivariable regression showed that age, grade of hypertension, residential area, exercise habits, eating speed, motivation to improve lifestyle, hypertension at the previous SHC, and the absence of a history or new onset of dyslipidemia were associated with the achievement of TBP < 140/90 mmHg. Consistent with trends reported in a previous study [12], more participants with lower grade and milder hypertension achieved TBP < 140/90 mmHg. Additionally, a higher proportion of participants who did not have hypertension at the previous SHC and had no record of a previous SHC tended to achieve TBP than those who also had hypertension at their previous SHC. Participants with mild hypertension or those who had hypertension for the first time or after some times were more likely to require less efforts for lifestyle modifications and fewer medications than those with more severe or persistent hypertension. We also focused on improving lifestyle as a possible intervention. Indeed, TBP achievement proportion was higher among participants with high motivation to improve their lifestyle, indicating the importance of promoting behavioral changes through medical care and health guidance for better BP control.

Various perspectives are relevant to differences in BP control status by place of residence. Hiratsuka City has a wide range of residents, from those who live in so-called urban areas to those who live in the countryside. In this context, we initially assumed that the number of medical facilities per place of residence would have a significant impact. Compared with area D, which has fewer medical facilities, especially in agricultural and industrial areas, area B is located near a train station and has more than twice as many medical facilities per 10,000 people, which was thought to be due to its easy accessibility. On the contrary, area A was a residential area near a central train station and did not contribute to TBP achievements despite the area having many medical facilities. One consideration is that the aging rate was lower than that in the other areas, which may have resulted in a lower health awareness. Area C also showed clear improvement despite having the same percentage of medical facilities as that in area D. This may be because area C has many commercial facilities and medical institutions located among them, and it also has good transportation access, which allows people to visit medical institutions efficiently.

The proportion of participants achieving TBP in this study was higher than that expected. Of importance, hypertension control in the US has deteriorated over the past 5 years, and measures to improve hypertension control have been reported [13, 14]. Strategies to be implemented at the individual, academic, community, and policy levels are presented, and at the community level, measures such as team-based care and lifestyle modification are used for assessment [15, 16]. One of the reasons for the high achievement proportion in this study is that Hiratsuka City has been proactive in providing SHG. This finding suggests that the city may have been more proactive in this area than in other areas, although SHG has little influence on BP control [16]. This study revealed the actual conditions of participants with hypertension at SHCs and the importance of promoting behavior change in lifestyle through medical care and health guidance to achieve antihypertensive goals. The findings will provide significant suggestions for future efforts in the community. In particular, approaches based on behavior science that differ from traditional methods should also be considered.

The limitation of this study is that BP measurements at medical institutions include white coat hypertension, which may cause discrepancy from home BP measurements. Additionally, the national average rate of health checkups is 50%, and selection bias cannot be ruled out because the participants were relatively health conscious. In this study, the achievement proportion was lower for the elderly. However, as the SHCs were limited to data for age ≤74 years, these data may not reflect the actual situation of the elderly with hypertension, including those aged ≥75 years. The low target achievement proportion suggests that the importance of antihypertensive treatment for elderly participants with hypertension may not have been fully understood in the medical field.

In conclusion, one-third of the Japanese adult participants who had hypertension failed to have BP control. For community-level management of hypertension, people who are motivated to improve their lifestyle, those relatively younger, those with mild hypertension, those without hypertension at the previous SHC, or those with no record of SHC in the previous year should be educated through public health nurses, which is an effective approach, while the elderly and those with persistent hypertension, as well as those with severe hypertension, should seek medical advice and continue lifestyle guidance in parallel. In terms of Asian Perspectives, since health guidance requires human resources, the results of this study may be helpful in considering that it is efficient to provide guidance based on priorities. In future, it could be essential to combine these efforts so that more efficient guidance can be tailored to the characteristics of each individual in this area because of cultural differences from other countries in Asia, represented by the more aged population in many countries and the consumption of a high-sodium diet.

## Supplementary information

Supplementary Figure and Table Legends

Table S1

Table S2

Figure S1

Figure S2
