# Supplementary material for: Modifiable factors to achieve target blood pressure in hypertensive participants
Source: Hypertens Res. 2025 Feb 19;48(4):1295–304. doi: 10.1038/s41440-025-02134-x (PMC11972950; doi:10.1038/s41440-025-02134-x)
Supplement: Supplementary file 1 — Supplementary Figure and Table Legends [file 41440_2025_2134_MOESM1_ESM.docx]

**Supplementary Summary**

Supplementary Table 1 provides additional data on the variables assessed.

Supplementary Table 2 provides information on the lifestyle-related behavior changes during analysis.

Supplementary Figures 1 and 2 provide unadjusted odds ratios for achieving target blood pressure <140/90 mmHg from the univariate and the multivariable logistical regression analysis, respectively.

**Supplementary Figure Legends**

**Supplementary Figure S1.** Results of univariate analysis of factors associated with achieving target blood pressure <140/90 mmHg

The unadjusted odds ratios for achieving TBP <140/90 mmHg were estimated in the univariable regression analysis of factors associated with achieving TBP <140/90 mmHg.

*Classification of high BP: grade I, SBP 140-159 mmHg and/or DBP 90-99 mmHg, grade II, SBP 160-179 mmHg and/or DBP 100-109 mmHg; grade III, SBP ≥180 mmHg and/or DBP ≥110 mmHg.

^†^Residential area A: located near the central train station, in a central residential area with relatively many commercial and public facilities, but not as many as B. There are also many medical institutions; area B: located near the train station, with a high number of commercial and public facilities, as well as medical institutions. Convenient transportation is also available; area C: located in the suburbs, with a high number of commercial facilities and fewer medical institutions. However, the medical institutions are mainly located in the commercial area; area D: located far away from the city center, with relatively many commercial and public facilities, but not as many as B. Transportation is inconvenient, and there are few medical institutions; area E: located far away from the city center, with few commercial facilities. Transportation is inconvenient, and there are few medical institutions.

Abbreviations: BP, blood pressure; CI, confidence interval; DBP, diastolic blood pressure; OR, odds ratio; SBP, systolic blood pressure; TBP, target blood pressure.

**Supplementary Figure S2.** Results of multivariable logistic regression analysis of factors associated with achieving target blood pressure <140/90 mmHg

The adjusted odds ratios for achieving TBP <140/90 mmHg were estimated using the multivariable logistic regression model including the following variables: sex; age; BMI; classification of high BP; residential area, history of diabetes, dyslipidemia, stoke, heart disease, CKD/renal failure, of anemia, hypertension at the previous SHC, new onset of diabetes, dyslipidemia, or CKD; and change in score after the index date to the next SHC for the variables smoking status, weight gain since the age of 20 years, exercise habits, physical activity, walking speed, chewing condition, eating speed, snacking, skipping breakfast, drinking status, alcohol consumption, sleep quality, interest in receiving SHG, motivation to improve lifestyle habits, and lifestyle score.

*Classification of high BP: grade I, SBP 140-159 mmHg and/or DBP 90-99 mmHg, grade II, SBP 160-179 mmHg and/or DBP 100-109 mmHg; grade III, SBP ≥180 mmHg and/or DBP ≥110 mmHg.

^†^Residential area A, located near the central train station, in a central residential area with relatively many commercial and public facilities, but not as many as B. There are also many medical institutions; area B, located near the train station, with a high number of commercial and public facilities, as well as medical institutions. Convenient transportation is also available; area C, located in the suburbs, with a high number of commercial facilities and fewer medical institutions. However, the medical institutions are mainly located in the commercial area; area D, located far away from the city center, with relatively many commercial and public facilities, but not as many as B. Transportation is inconvenient, and there are few medical institutions; area E, located far away from the city center, with few commercial facilities. Transportation is inconvenient, and there are few medical institutions.

Abbreviations: BMI, body mass index; BP, blood pressure; CKD, chronic kidney disease; CI, confidence interval; DBP, diastolic blood pressure; OR, odds ratio; SBP, systolic blood pressure; SHC, specific health checkup; SHG, specific health guidance TBP, target blood pressure.
