# Supplementary material for: Modifiable factors to achieve target blood pressure in hypertensive participants
Source: Hypertens Res. 2025 Feb 19;48(4):1295–304. doi: 10.1038/s41440-025-02134-x (PMC11972950; doi:10.1038/s41440-025-02134-x)
Supplement: Supplementary file 2 — Table S1 [file 41440_2025_2134_MOESM2_ESM.docx]

**Supplementary Table S1.** Definition of extracting variables

|  | SHC results | List of insured persons | Claims database | ICD-10 code* | Drug code^†^ | Drug code name | Laboratory values |
| --- | --- | --- | --- | --- | --- | --- | --- |
| Extraction period: Baseline period |  |  |  |  |  |  |  |
| Hypertension therapy | ● |  | ● | I10-15 | 2123, 2132-2135, 2139, 2142-2145, 2149, 2171, 2190 | beta-blockers, thiazides, anti-aldosterone products, carbonic anhydrase inhibitors, chlorobenzenesulfonamide derivatives and preparations, other diuretics, hydralazine and preparations, rauwolfia preparation, angiotensin-converting enzyme inhibitors, methyldopa and preparations, other antihypertensives, coronary vasodilators, miscellaneous cardiovascular agents |  |
| History of diabetes | ● |  | ● |  | 2492, 2499, 3961, 3962, 3969 | pancreatic hormones, hormonal products not elsewhere classified, sulfonylureas, biguanides, other agents for diabetes |  |
| History of dyslipidemia | ● |  | ● |  | 2183, 2189, 2190, 3399 | clofibrate derivative, other antihyperlipidemic agents, miscellaneous CV agents, blood and body fluid agents-miscellaneous |  |
| History of stroke |  |  | ● | I60-64, I690, I691, I693, I694, G45 |  |  |  |
| History of heart disease |  |  | ● | I20-22, I42, I48, I50, I517 |  |  |  |
| History of CKD/renal failure | ● |  | ● | N17-19 |  |  | eGFR<60 |
| History of anemia | ● |  | ● | D50-64 |  |  | male Hb<13, female Hb<12 |
| Extraction period: Index date and next SHC |  |  |  |  |  |  |  |
| Sex | ● |  |  |  |  |  |  |
| Age | ● |  |  |  |  |  |  |
| Waist circumference | ● |  |  |  |  |  |  |
| BMI | ● |  |  |  |  |  |  |
| BP | ● |  |  |  |  |  |  |
| Urine protein | ● |  |  |  |  |  |  |
| HbA1c (NGSP) | ● |  |  |  |  |  |  |
| BS (fasting) | ● |  |  |  |  |  |  |
| TG | ● |  |  |  |  |  |  |
| LDL | ● |  |  |  |  |  |  |
| AST | ● |  |  |  |  |  |  |
| ALT | ● |  |  |  |  |  |  |
| γ-GTP | ● |  |  |  |  |  |  |
| eGFR | ● |  |  |  |  |  |  |
| Interest of receiving health guidance | ● |  |  |  |  |  |  |
| Motivation to improve lifestyle habits | ● |  |  |  |  |  |  |
| Residential area |  | ● |  |  |  |  |  |
| Smoking status | ● |  |  |  |  |  |  |
| Weight gain since the age of 20 years | ● |  |  |  |  |  |  |
| Exercise habits | ● |  |  |  |  |  |  |
| Physical activity | ● |  |  |  |  |  |  |
| Walking speed | ● |  |  |  |  |  |  |
| Chewing condition | ● |  |  |  |  |  |  |
| Eating speed | ● |  |  |  |  |  |  |
| Late-night eating | ● |  |  |  |  |  |  |
| Snacking | ● |  |  |  |  |  |  |
| Skipping breakfast | ● |  |  |  |  |  |  |
| Drinking status | ● |  |  |  |  |  |  |
| Alcohol consumption | ● |  |  |  |  |  |  |
| Sleep quality | ● |  |  |  |  |  |  |
| Interest in receiving health guidance | ● |  |  |  |  |  |  |
| Lifestyle score | ● |  |  |  |  |  |  |
| Extraction period: Previous SHC |  |  |  |  |  |  |  |
| Hypertension at last SHC | ● |  |  |  |  |  |  |
| Extraction period: After index date to next SHC |  |  |  |  |  |  |  |
| Receiving SHG | ● |  |  |  |  |  |  |
| New onset of diabetes |  |  | ● |  | 2492, 2499, 3961, 3962, 3969 |  |  |
| New onset of dyslipidemia |  |  | ● |  | 2183, 2189, 2190, 3399 |  |  |
| New onset of CKD |  |  | ● | N18 |  |  |  |
| CV events |  |  | ● | I20-22, I50, I60-64, I690, I691, I693, I694, G45 |  |  |  |

Abbreviations: ALT, alanine aminotransferase; AST, aspartate transaminase; ATC, Anatomical Therapeutic Chemical; BMI, body mass index; BP, blood pressure, BS, blood sugar: CKD, chronic kidney disease; eGFR, estimated glomerular filtration rate; GTP, guanosine triphosphate; HbA1c, hemoglobin A1c; ICD, International Classification of Disease; LDL, low-density lipoprotein; NGSP, National Glycohemoglobin Standardization Program; SHC, specific health checkup; SHG, specific health guidance; TG, triglyceride.

*Version for 2013

^†^Standardized drug codes managed by Health Insurance Claims Review & Reimbursement Service, which listed individual prescription drugs in Japan, based on the versions from December 8, 2022. The code consists of 12 digits: 4 digits (therapeutic category number) + 3 digits (route of administration + active ingredient) + 1 digit (dosage form) + 1 digit (different specification) + 2 digits (brand name) + 1 digit (check digit). The first 4 digits were used for the definition.
