# Supplementary material for: Modifiable factors to achieve target blood pressure in hypertensive participants
Source: Hypertens Res. 2025 Feb 19;48(4):1295–304. doi: 10.1038/s41440-025-02134-x (PMC11972950; doi:10.1038/s41440-025-02134-x)
Supplement: Supplementary file 3 — Table S2 [file 41440_2025_2134_MOESM3_ESM.docx]

**Supplementary Table S2.** Changes in lifestyle-related behaviors after the index date until the next specific health checkup and the number of participants achieved target blood pressure <140/90 mmHg

| **Variable** |  | **Number of participants** | **Number of participants achieved** | **Number of participants not achieved** |
| --- | --- | --- | --- | --- |
| Smoking status | improved | 58 (1.1%) | 37 (63.8%) | 21 (36.2%) |
|  | no change | 5346 (98.5%) | 3119 (58.3%) | 2227 (41.7%) |
|  | worsened | 24 (0.4%) | 15 (62.5%) | 9 (37.5%) |
| Weight gain since the age of 20 | improved | 286 (5.3%) | 169 (59.1%) | 117 (40.9%) |
|  | no change | 4871 (90.1%) | 2836 (58.2%) | 2035 (41.8%) |
|  | worsened | 250 (4.6%) | 156 (62.4%) | 94 (37.6%) |
| Exercise habits | improved | 546 (10.1%) | 291 (53.3%) | 255 (46.7%) |
|  | no change | 4405 (81.6%) | 2593 (58.9%) | 1812 (41.1%) |
|  | worsened | 449 (8.3%) | 273 (60.8%) | 176 (39.2%) |
| Physical activity | improved | 630 (11.7%) | 354 (56.2%) | 276 (43.8%) |
|  | no change | 4212 (78%) | 2487 (59%) | 1725 (41%) |
|  | worsened | 555 (10.3%) | 315 (56.8%) | 240 (43.2%) |
| Walking speed | improved | 461 (8.6%) | 263 (57%) | 198 (43%) |
|  | no change | 4460 (82.9%) | 2605 (58.4%) | 1855 (41.6%) |
|  | worsened | 459 (8.5%) | 275 (59.9%) | 184 (40.1%) |
| Chewing condition | improved | 337 (6.2%) | 194 (57.6%) | 143 (42.4%) |
|  | no change | 4708 (87.2%) | 2750 (58.4%) | 1958 (41.6%) |
|  | worsened | 351 (6.5%) | 209 (59.5%) | 142 (40.5%) |
| Eating speed | improved | 383 (7.1%) | 221 (57.7%) | 162 (42.3%) |
|  | no change | 4559 (84.9%) | 2669 (58.5%) | 1890 (41.5%) |
|  | worsened | 429 (8%) | 239 (55.7%) | 190 (44.3%) |
| Late-night eating | improved | 324 (6%) | 207 (63.9%) | 117 (36.1%) |
|  | no change | 4799 (89%) | 2787 (58.1%) | 2012 (41.9%) |
|  | worsened | 267 (5%) | 156 (58.4%) | 111 (41.6%) |
| Snacking | improved | 728 (13.5%) | 434 (59.6%) | 294 (40.4%) |
|  | no change | 3923 (72.6%) | 2258 (57.6%) | 1665 (42.4%) |
|  | worsened | 750 (13.9%) | 461 (61.5%) | 289 (38.5%) |
| Skipping breakfast | improved | 128 (2.4%) | 78 (60.9%) | 50 (39.1%) |
|  | no change | 5133 (95.2%) | 2993 (58.3%) | 2140 (41.7%) |
|  | worsened | 130 (2.4%) | 75 (57.7%) | 55 (42.3%) |
| Drinking status | improved | 369 (6.8%) | 239 (64.8%) | 130 (35.2%) |
|  | no change | 4727 (87.6%) | 2739 (57.9%) | 1988 (42.1%) |
|  | worsened | 301 (5.6%) | 177 (58.8%) | 124 (41.2%) |
| Alcohol consumption | improved | 331 (6.1%) | 188 (56.8%) | 143 (43.2%) |
|  | no change | 4796 (88.9%) | 2807 (58.5%) | 1989 (41.5%) |
|  | worsened | 269 (5%) | 159 (59.1%) | 110 (40.9%) |
| Sleep quality | improved | 366 (6.9%) | 216 (59%) | 150 (41%) |
|  | no change | 4550 (85.6%) | 2667 (58.6%) | 1883 (41.4%) |
|  | worsened | 397 (7.5%) | 220 (55.4%) | 177 (44.6%) |
| Interest in receiving SHG | improved | 412 (7.7%) | 235 (57%) | 177 (43%) |
|  | no change | 4436 (82.6%) | 2588 (58.3%) | 1848 (41.7%) |
|  | worsened | 524 (9.8%) | 314 (59.9%) | 210 (40.1%) |
| Willingness to improve lifestyle habits | improved | 929 (17.4%) | 575 (61.9%) | 354 (38.1%) |
|  | no change | 3602 (67.4%) | 2095 (58.2%) | 1507 (41.8%) |
|  | worsened | 814 (15.2%) | 448 (55%) | 366 (45%) |
| Lifestyle score | improved | 630 (12.1%) | 355 (56.3%) | 275 (43.7%) |
|  | no change | 4022 (77.2%) | 2367 (58.9%) | 1655 (41.1%) |
|  | worsened | 558 (10.7%) | 321 (57.5%) | 237 (42.5%) |

Data are presented as numbers and percentages.
