# Supplementary figures and images for: Modifiable factors to achieve target blood pressure in hypertensive participants
Source: Hypertens Res. 2025 Feb 19;48(4):1295–304. doi: 10.1038/s41440-025-02134-x (PMC11972950; doi:10.1038/s41440-025-02134-x)

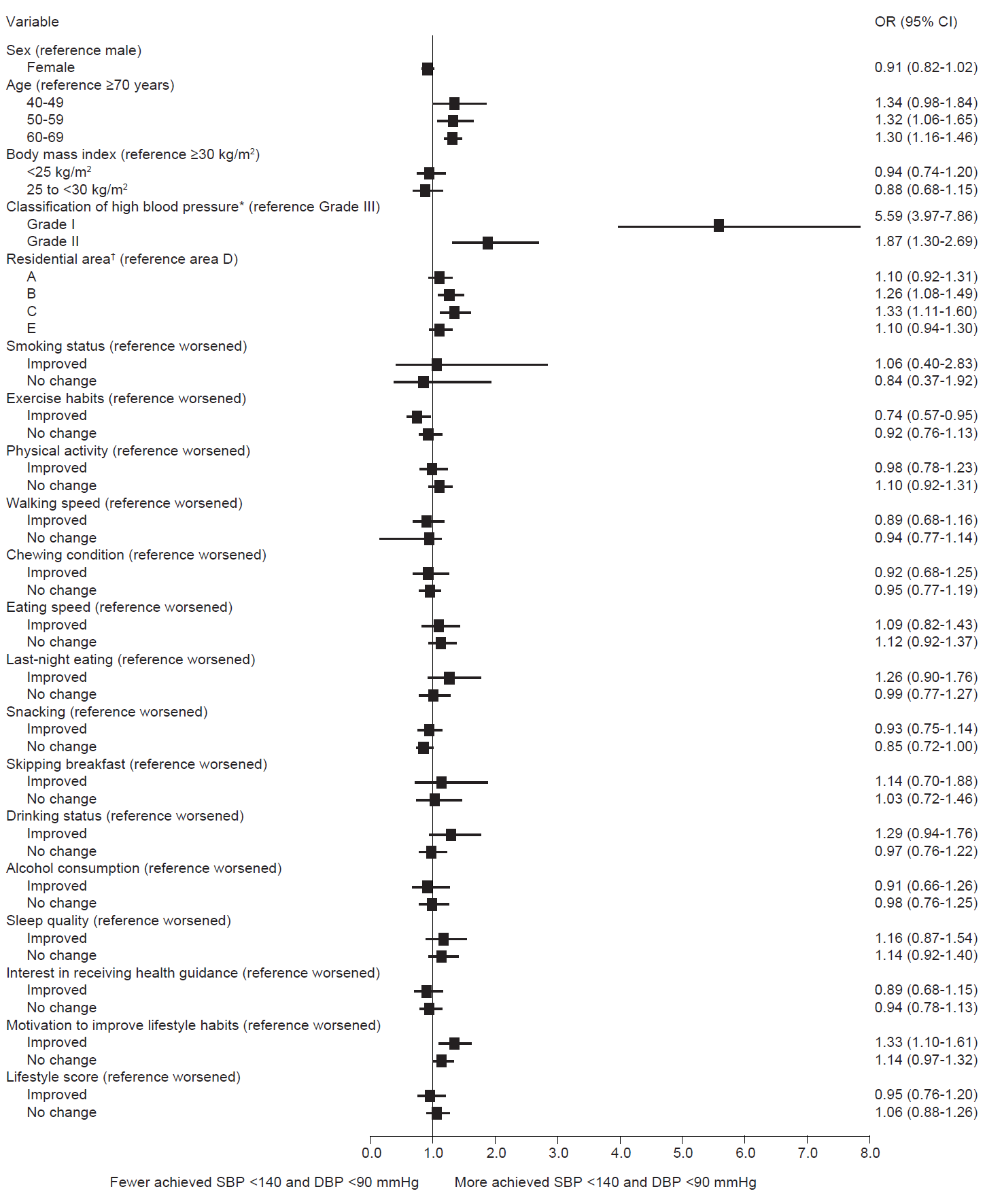
**Supplement Figure 1**


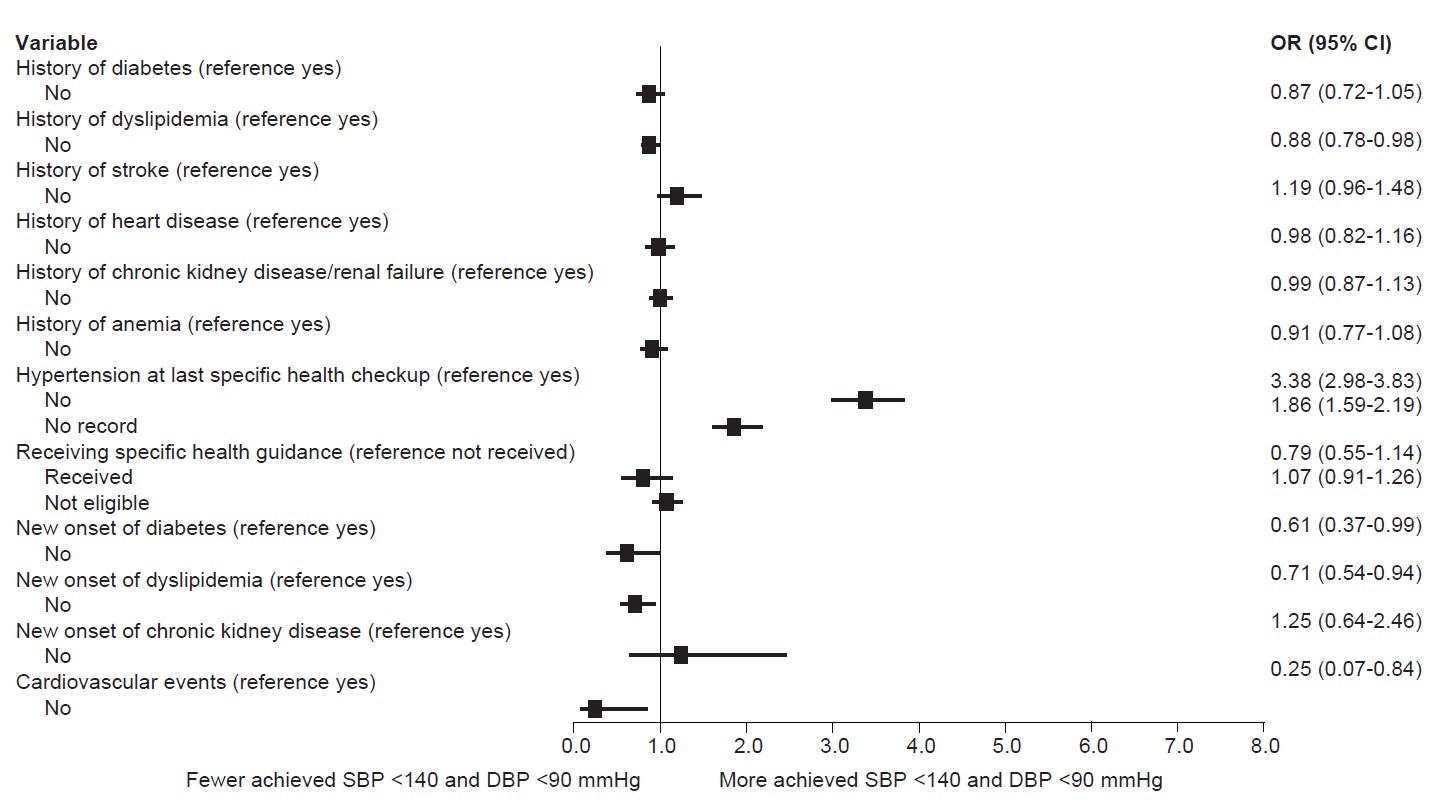

Supplement: Supplementary file 4 — Figure S1 [file 41440_2025_2134_MOESM4_ESM.docx]

**Supplement Figure 2**


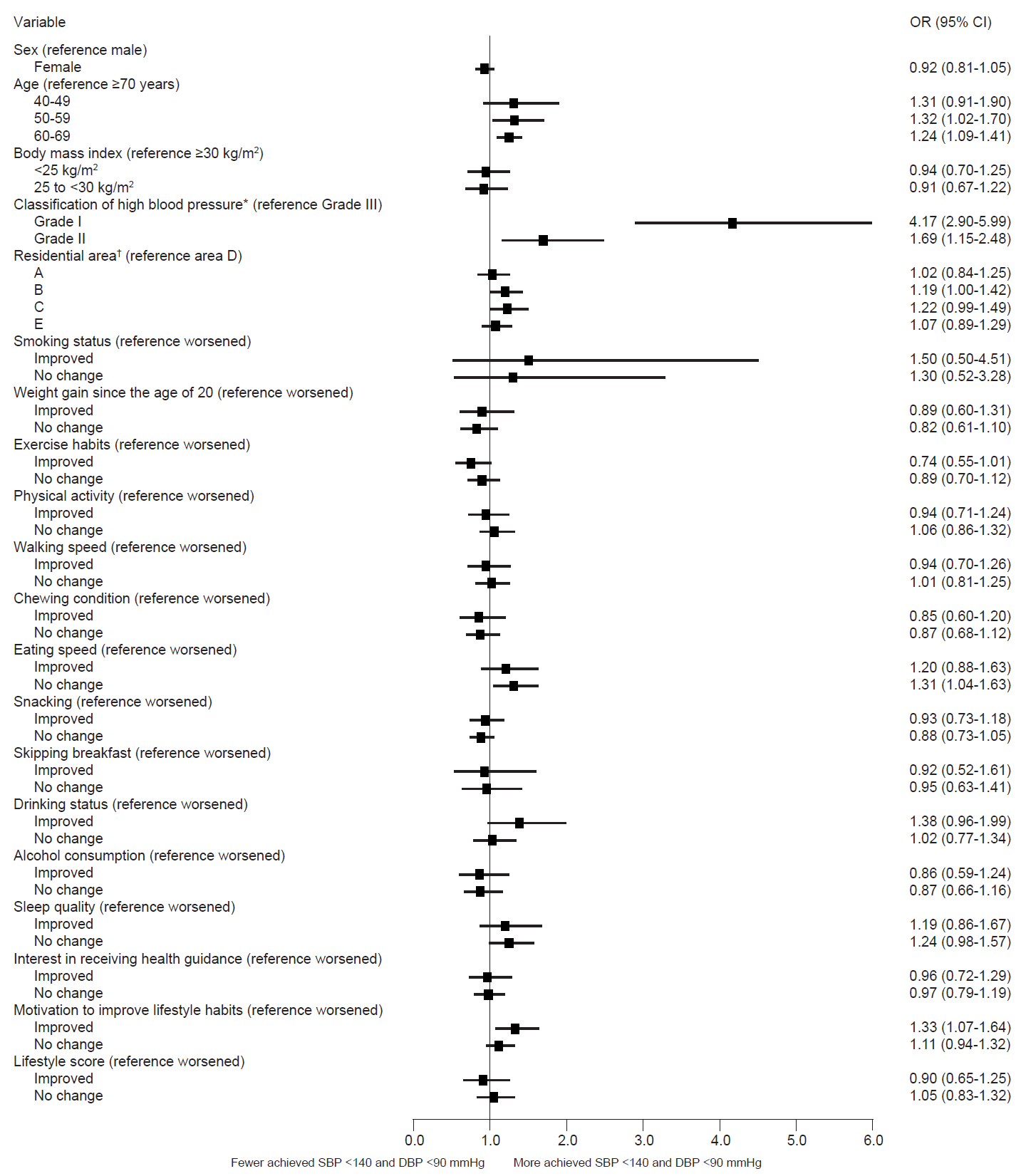


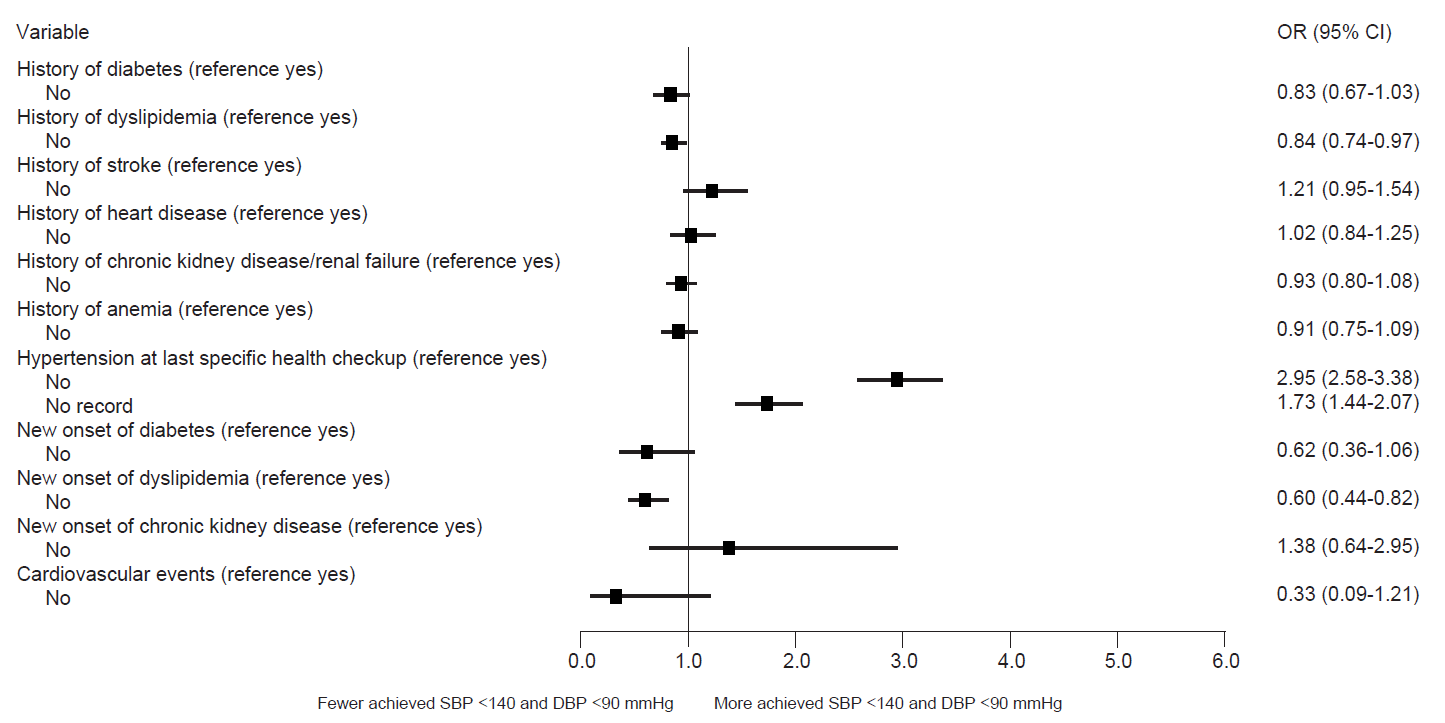

Supplement: Supplementary file 5 — Figure S2 [file 41440_2025_2134_MOESM5_ESM.docx]
